# Auditory Processing and Speech-Sound Disorders

**DOI:** 10.3390/brainsci14030291

**Published:** 2024-03-19

**Authors:** Konstantinos Drosos, Alexandra Papanicolaou, Louiza Voniati, Klea Panayidou, Chryssoula Thodi

**Affiliations:** 1 School of Sciences, Speech and Language Pathology, European University Cyprus, Nicosia 2404, Cyprus; k.drosos@external.euc.ac.cy (K.D.); l.voniati@euc.ac.cy (L.V.);; 2 Department of Hearing and Speech Sciences, University of Maryland College Park, College Park, MD 20740, USA; apapani3@umd.edu

**Keywords:** auditory processing, speech-sound disorder, language, assessment, comorbidity, phonological awareness

## Abstract

Background: Speech-sound disorders (SSD) have been linked to auditory processing difficulties, and auditory processing disorders (APD) have been related to phonological awareness and literacy development. To this date, there has not been a systematic literature review investigating the results of psychophysiology and language assessments related to SSD and APD in children. Methods: The literature search was conducted in PubMed, Medline EBSCO, and Scopus to identify studies with children diagnosed/suspected of having APDs and SSDs. The quality of methodology in the selected articles was evaluated with the Newcastle Ottawa Scale. Results: Seven out of 378 relevant studies met the selection criteria. The findings were summarized for children with SSD and APD based on (a) metalinguistic and literacy skills, (b) cognitive abilities, and (c) temporal processing abilities. Three articles indicated that children with APD and SSD exhibit lower temporal task accuracy and reaction time. In two studies, children with SSD exhibited lower scores in discrimination, sequencing, and recall of brief stimuli in rapid succession. Conclusions: This review revealed associations between SSD severity and APD that may underline low performance in metalinguistic skills. Diagnostic assessments have been proposed based on the review to adequately identify children with SSD and APD and provide useful information for more suitable intervention.

## 1. Introduction

Auditory processing (AP) is defined as the perceptual processing of auditory information in the central nervous system and the neurobiological activity that underlies that processing and gives rise to the electrophysiologic auditory potentials [1]. AP involves the auditory mechanisms responsible for discrimination of speech and non-speech signals, sound localization and lateralization, auditory pattern recognition, temporal aspects of audition, and auditory performance with degraded and competing acoustic signals [2,3,4,5,6,7]. The critical roles of auditory processing in communication, speech [8,9], and cognitive development [10] have been highlighted in the literature. The American Speech and Hearing Association (ASHA) and the American Academy of Audiology (AAA) indicate that various factors affect auditory processing and language development in children, including chronic otitis media, inheritable factors, auditory neuropathy, and genetic and environmental influences [3,6]. Auditory processing disorder (APD) is a deficit in the neural processing of auditory information that can lead to higher-order disorders related to learning, attention, memory, and cognitive-, communicative-, or language-related skills [3,6]. The prevalence of reported APD varies widely, from 0.5 to 10% of the general population [11,12,13]. In children, the estimated prevalence is between 0.2 and 5% [13,14,15], noted as a 2:1 male-to-female ratio of APD diagnoses.

A wide range of sensory, cognitive, social, and language skills are associated with the development and functionality of auditory processing. Several studies suggest high comorbidity between APD and developmental disorders, such as specific language impairment (SLI), dyslexia, learning disorder (LD), attention-deficit hyperactivity disorder (ADHD), and autism spectrum disorders (ASD) [16,17,18]. Systematic reviews examining characteristics of APD and influences of non-auditory factors in school-aged children with APD reveal that children with APD experience multimodal difficulties in specific language areas. These challenges primarily manifest in phonological awareness and literacy skills [15,19]. The results align with research by Leite, Wertzner, and Matas [20], which also highlighted heightened challenges in phonological development among children with APD. The integrity of auditory processing plays a critical role in proper phonological development, as failure or interference in the cortical processing of auditory information is expected to affect the integration, understanding, and interpretation of sound [20]. 

Auditory temporal processing skills help identify temporal elements that differentiate various speech sounds and determine speech intelligibility [3]. Auditory temporal resolution is critical for speech perception and successful language development in children [21]. Difficulty in temporal processing could interfere with the phonemic representation in the brain leading to speech-sound disorders (SSD) [22,23]. Children with APD struggle with auditory closure, temporal processing, and auditory memory [24]. Difficulty in recognizing rapid temporal acoustic cues influences phoneme identification and aspects related to speech recognition [25]. Sayyahi et al. reported a significant relationship between the gap-detection threshold and speech error consistency in children with SSD [21]. Muluk et al. found poorer gap-detection performance in children with previous language delay and SSD [26]. Moreover, children with SSD performed poorly in auditory and visual sustained-attention tasks, such as gap detection and frequency pattern discrimination tasks [27].

A relationship between APD and SSD has been postulated based on the premise that disruption in processing auditory parameters interferes with the stability of phonemic representation in the brain and with speech perception and, hence, complicates morphology learning [28]. It can be challenging to independently study AP in children with SSD, given that speech-production output and cognitive–linguistic deficits lead to phonological processing difficulties at the phonemic level. Many studies support that APD may be one of the major underlying factors in SSD, as lower scores in auditory processing tasks are often observed in children with varying SSD [9,29,30,31,32,33,34].

The age at which speech-sound disorders can be clinically diagnosed is between 3 and 6 years; many children with suspected SSD are referred to speech and language therapy before age four. APD assessment is conducted primarily after the age of 7 years [6]. Given similar behaviors and characteristics, comorbid disorders are often diagnosed in place of or in tandem with APD [8,35]. Parsing purely auditory from cognitive disorders is challenging both theoretically and operationally, as many listening tasks use complex stimuli such as speech and noise to reach a diagnosis [36,37]. 

There is a considerable corpus of research relating to language and auditory processing, mainly focused on phonological skills and phonological awareness. To our knowledge, there is no comprehensive literature review encompassing all the available literature sources on the topic of APD and SSD. A systematic review published by Pereira et al. attempted to characterize the impact of APD in children with phonological disorders; however, the analysis of the pertinent literature was limited to one article [38]. Furthermore, the identification of assessment protocols and clinical and/or objective auditory indicators to guide appropriate evaluation, diagnosis, and individualized management of auditory processing disorder remain heavily discussed in the available literature [9,30]. Detection of APD and SSD comorbidity along with the development of appropriate assessment protocols for both APD and SSD can be clinically helpful for audiologists, speech pathologists, and other practitioners [19,37]. Hence, this systematic review aims to investigate comorbidities and interactions of auditory processing in children with SSD, in addition to identifying appropriate testing material to specify diagnosis and to individualize intervention. 

## 2. Materials and Methods

Between December 2022 and December 2023, systematic literature research was conducted in three databases, PubMed, Medline EBSCO, and Scopus Elsevier, to identify studies published until December 2023 using specific keywords, as indicated in Table 1. The population, intervention, comparison, outcome (PICO) framework was used to develop the clinical questions for our systematic review. To support this framework, our clinical question is focused on (a) identifying children with suspected or diagnosed APD and children diagnosed with SSD (P), (b) evaluating children’s diagnoses and observing children’s performances on auditory, speech, language, and other secondary tasks (I), (c) comparing characteristics and performance between children with APD and SSD (C), and (d) specifying diagnostic measures and clinical characteristics amongst this sub-population to ensure accurate diagnosis and intervention (O). This scoping review was reported according to the Preferred Reporting Items for Systematic Reviews and Meta-Analyses extension for Scoping Reviews (PRISMA-ScR) [39]. Studies related to APD and SSD according to diagnostic criteria established by AAA and ASHA. The methodological quality analysis of the identified papers was done according to the New Castle Ottawa criteria [40].

Researchers deemed studies published between 1990 and December 2023 eligible for review. The selection criteria included the following: (a) the study must be written in English and published in a peer-reviewed journal, (b) the study should contain data related to participants under the age of 18 years old, and (c) the study must include children with suspected or clinically diagnosed APD and children diagnosed with SSD according to DSM-V criteria. Studies including children with peripheral hearing loss, chronic otitis media, brain damage, neuropathy, mental or developmental disorders (i.e., learning difficulties (LD), specific language impairment (SLI), dyslexia, attention-deficit hyperactivity disorder (ADHD), or autism spectrum disorder (ASD), and syndromes), and cochlear implants recipients were excluded from the review.

**Table 1:** Data from identification of studies in databases.

Databases	Keywords
PubMed	(((AUDITORY PROCESSING [Title/Abstract] OR APD [Title/Abstract] OR AUDITORY PROCESSING DISORDER [Title/Abstract])) AND (PHONOLOGICAL AWARENESS [Title/Abstract] OR SPEECH SOUND DISORDER [Title/Abstract])) NOT (DYSLEXIA [Title/Abstract] OR LEARNING DISABILITIES [Title/Abstract]) NOT (ADULTS [Title/Abstract] OR AUTISM [Title/Abstract])
Medline EBSCO	AB (AUDITORY PROCESSING OR APD OR AUDITORY PROCESSING DISORDER) AND AB (PHONOLOGICAL AWARENESS OR SPEECH SOUND DISORDER) NOT AB (DYSLEXIA OR LEARNING DISABILITIES) NOT AB (ADULTS NOT AB AUTISM)
Scopus Elsevier	(TITLE-ABS-KEY (AUDITORY AND PROCESSING OR APD OR AUDITORY AND PROCESSING AND DISORDER) AND TITLE-ABS-KEY (PHONOLOGICAL AND AWARENESS OR SPEECH AND SOUND AND DISORDER) AND NOT TITLE-ABS-KEY (DYSLEXIA OR LEARNING AND DISABILITIES) AND NOT TITLE-ABS-KEY (ADULTS) AND NOT TITLE-ABS-KEY (AUTISM))

## 3. Results

This systematic review included studies on children with typical psychomotor development evaluated with language, auditory processing, and behavioral tests according to ASHA and AAA [3,6] recommendations. Three hundred eighty-four studies were compiled in the initial search with keyword combinations; 244 studies were excluded as duplicates. The remaining 137 articles were screened to assess compliance with the inclusion criteria regarding participants, disorders, and methodology. 

**Figure 1:**
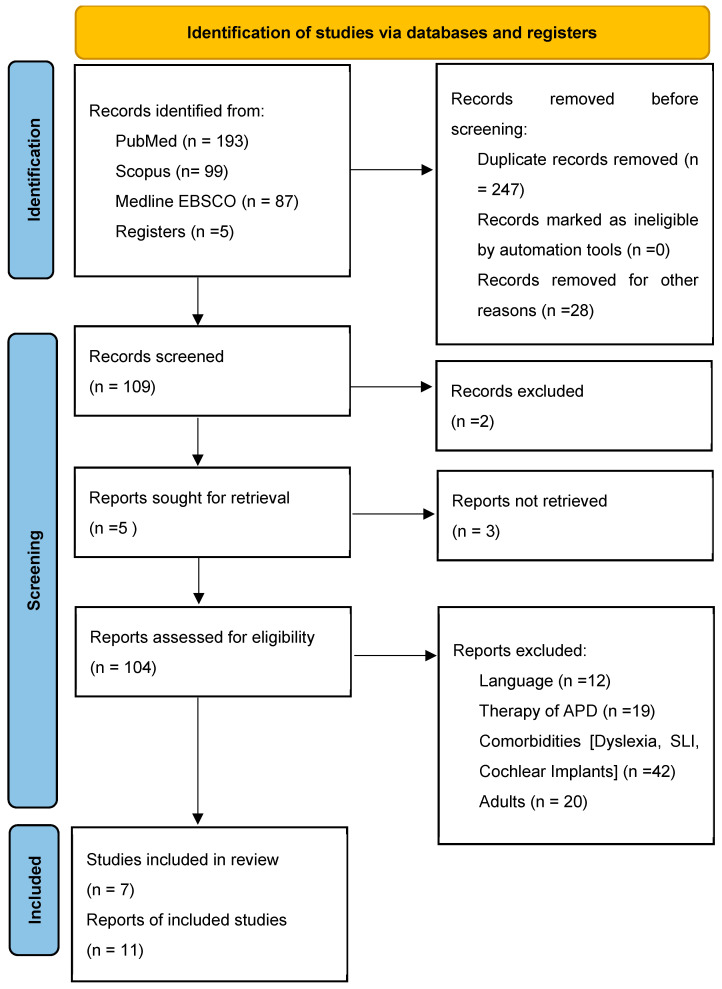
Prisma flow diagram [39].

As illustrated in Figure 1, 12 articles were excluded due to the language of publication; 114 articles were excluded for reasons related to study methodology (e.g., case report) and participant characteristics (e.g., participants with other comorbidities). Seven studies were selected for the full-length systematic review, three of which were published in the last five years [41,42,43], and four of which were published in the last decade [44,45,46,47]. An additional four articles were included in the analysis, but their discussion was only partially reviewed due to ineligible study design (such as pilot studies) and title incompatibility. The studies included in this systematic review were analyzed and rated according to the Newcastle Ottawa Scale (NOS) for case-control studies by two reviewers (illustrated in Table 2).

The outcomes of this systematic review are summarized with respect to three areas: language assessment (Table 3), behavioral assessment (Table 4), and auditory processing assessment (Table 5), including the articles that were partially reviewed. The studies that fit the inclusion criteria reported findings on various tests and presented these findings in multiple ways. The studies used different assessment procedures and evaluation methods due to language differences and differences in standardized test batteries across languages. Beyond this variability of reported findings, all studies provided helpful information on auditory processing and the brain’s ability to perceive and encode linguistic and non-linguistic information.

### 3.1. Study Quality Assessment 

Study quality assessment for the included studies was made according to the Newcastle Ottawa Scale (NOS). The maximum quality score for each study is nine points. The score for the studies included in the systematic review was between 7 and 8 points. The NOS scores between authors’ assessments were compared with the Wilcoxon paired sign-rank test, as described in Lo et al. [48]. The results indicated that the mean rating scales were equal between authors across the articles reviewed in the analysis (*p* ≤ 0.05). No blindness for the researchers or the participants was stated. Good homogeneity is recorded as it relates to the criteria for diagnosis, sample selection, and the separation of the experimental and control groups. It is noted that none of the studies presented detailed information on the participants’ withdrawals.

### 3.2. Speech, Language, and Cognitive Abilities in Children with APD

Sharma et al. [44] investigated 68 children between the ages of 7 to 12 years old (mean = 9.75 years old), including 44 boys and 24 girls. Participating children had no prior formal diagnoses other than APD (*n* = 9) and, in some cases, reading disorder (RD) (*n* = 20). Participants either had a suspicion of APD as reported by their parents or teachers or had been diagnosed with APD by an audiologist, a speech-language therapist, and/or an educational psychologist. These children completed a series of tests on auditory processing, phonological awareness, language, reading, and writing skills. Five behavioral tests were used to test a range of auditory processes and identify children with APD: (a) Dichotic Digit Test Version 2 (DDT-2) [49], (b) Frequency Pattern Test (FPT) [50,51], (c) Random Gap Detection Test (RGDT) [52], (d) compressed (45%) and reverberant (0.3-s) CVC words [53], and (e) 500-Hz tone Masking Level Difference (MLD) [54]. The Queensland University Inventory of Literacy (QUIL) [55] was used for the phonological awareness assessment. Forty-nine (72%) children were diagnosed with APD, and nearly half (47%) of them had problems in all three areas tested (AP, language, and reading). Of these children, only 4% had APD in isolation. Among the 49 children diagnosed with APD, most children (82%) scored poorly on the FPT in both ears, and more than half (65%) of these children had phonological awareness difficulties. The FPT scores were best for all children who did not have APD (LI, RD, and RD-LI) and the poorest for those who had APD with reading delay (RD) and/or language impairment (LI). The authors highlighted pronounced comorbidities between APD–LI–RD and indicated the necessity for assessment of all three areas when evaluating children with suspected APD, to specify diagnosis and personalize interventions.

**Table 2:** Data from Newcastle-Ottawa Quality Assessment Scale Rating (Reviewer 1/Reviewer 2). Each asterisk represents if an individual criterion within the subsection was fulfilled. ~ means no record.

	Quality Assessment Criteria	Acceptable (*) (Reviewer 1/Reviewer 2)	Sharma et al. [36]	Allen and Allan [45]	Barrozo et al. [46]	Vilela et al. [47]	Lam et al. [43]	Jung and Lee [42]	Assis et al. [41]	Jain et al. [33]	Attoni et al. [54]	Murphy et al. [27]	Vilela et al. [56]
Selection	Is the case definition adequate?	AP and PA tests and History	*/*	*/*	*/*	*/*	*/*	*/*	*/*	*/*	*/*	*/*	*/*
	Representativeness of the cases?	From the same group or by the test results	*/*	*/*	*/*	*/*	*/*	*/*	*/*	*/*	*/*	*/*	*/*
	Selection of controls?	From the same group	*/*	*/*	*/*	*/*	*/*	*/*	*/*	*/*	*/*	*/*	*/*
	Definition of controls?	From tests and History	~/~	*/*	*/*	*/*	*/*	*/*	*/*	*/*	*/*	*/*	*/*
Comparability													
	Comparability of cases and controls on the basis of the design or analysis.	Matched for Age and Desing	**/**	*/*	**/**	*/*	**/**	*/*	**/**	*/*	*/*	**/**	*/*
Exposure													
	Ascertainment of exposure.	Structured data by tests and History files	*/*	*/*	*/*	*/*	*/*	*/*	*/*	*/*	*/*	*/*	*/*
	Same method of ascertainment for cases and controls.	Same assessments for all subjects	*/*	*/*	*/*	*/*	*/*	*/*	*/*	*/*	*/*	*/*	*/*
	Non-response rate.	No designation	~	~	~	~	~	~	~	~	~	~	~
Overall Quality Score			7/7	8/7	8/8	8/7	8/8	8/7	8/8	7/7	7/7	8/8	7/7

Allen and Allan [45] investigated 63 children with suspected APD between the ages of 7 and 17 years, with low academic performance and no other reported disorder. The authors compared phonology, memory, and auditory processing performance between children identified with APD and those with no APD findings. Thirty-nine children were male, and 24 were female. The children completed a battery of linguistic, behavioral, and audiological tests (subjective and objective in nature). Forty of the 63 children were subsequently classified as APD based on the behavioral test battery. Of the children diagnosed with APD, 15 failed two tests, 13 failed three tests, 10 failed four tests, and two failed all tests. The remaining 23 did not receive a formal diagnosis of APD. Ten of the 23 children not diagnosed with APD scored within expectations on all tests, and 13 scored below expectations on only one test. Children with APD had lower scores on behavioral and language assessments than children without APD. The Gap Detection Task (AFT-R) revealed poor performance amongst most children followed by the Dichotic Staggered Spondee Word (SSW) test. Children classified as APD generally performed more poorly than those without APD in the overall assessment of intelligence and academic performance. Furthermore, the performance on listening comprehension, oral expression, and written language derived from the Oral and Written Language Scales (OWLS) was significantly poorer amongst children with APD. The results also reflected poorer phonological performance in the group of children with APD on the Comprehensive Test of Phonological Processing (CTOPP) test. Objective auditory measures were also obtained based on acoustic reflex thresholds (ART) and auditory brainstem responses (ABR). The authors found that many children, independent of APD diagnosis, had abnormal findings on ART and on one or more ABR waveform measures. These findings suggested poor auditory processing may be related to abnormal auditory brainstem function. Based on the examinations obtained from behavioral audiological skills, cognitive performance, and measures of auditory neural integrity, the authors concluded that test batteries that are limited to behavioral speech-based tests alone may lead to misdiagnosis of APD when the primary deficit may be language-related instead. 

Lam et al. [43] aimed to explore the effects of auditory processing deficits on reading and cognitive processes. Frequency discrimination (FD) was the auditory processing ability evaluated in this study, given its strong relationship to reading skills. In total, 16 children with APD (aged between 7 and 11 years) were assessed using a computer-based (FD) game, Sounds Pod. Eight children had age-appropriate performance (FD-typical group) and the other eight performed poorly on the task (FD-poor group). All children were further assessed on lexical and non-lexical reading, phonological processing (phonological awareness, phonological memory, and rapid automizing naming), receptive language, auditory sustained attention, and executive function. Non-lexical and lexical reading was evaluated using the Assessment of Lexical and Non-Lexical Reading Abilities in Children (ALNLRAC), phonological processing was assessed using the CTOPP, and receptive language was assessed using the Peabody Picture Vocabulary Test (PPVT). The FD-poor group showed significantly poorer performance on non-lexical reading, phonological awareness, and executive function. The authors postulated that executive function may contribute to poor performance on perceptual tasks, such as FD and reading abilities. However, the study showed a stronger association between FD performance, reading difficulty, and phonological awareness in children with APD. These findings support the notion that non-lexical reading and phonological awareness skills are dependent on auditory processing abilities. 

### 3.3. Speech, Language, and Cognitive Abilities in Children with SSD

Assis et al. [41] compared the auditory processing performance in children with and without a phonological disorder (PD) through a word–picture identification task using the speech-perception instrument, PERCEFAL. The instrument included auditory stimuli comprising of recorded target words and their minimal pair and visual stimuli comprising pictures that directly correspond to each word. Forty-six children were included in the study, aged 4 and 8 years old, 23 of whom were diagnosed with a PD with involvement in the stops class (Group 1), and 23 typically developed children (Group 2). Data regarding the auditory perceptual performance of the stops were obtained, including the reaction time, number of errors and correctness, and pattern of perceptual error. Correctness was defined as auditory perceptual accuracy measured through correct production of target words The researchers found significant differences in the percentage of errors and correctness as well as in the reaction time related to the correctness between the two groups. Mainly, children with PD had higher error rates and longer reaction times related to correctness. The longer reaction time observed in children with PD for correctness may suggest a greater demand for properly processing speech. Overall, the results found in this study suggest that children with PD may also have a speech-perception deficit, implying that perceptual deficits could interfere or interact with production deficits. 

Attoni, Quintas, and Motta [54] assessed auditory processing and phonemic discrimination in children with and without phonological disorders (PD). The secondary aim was to correlate acoustic reflex thresholds (ART) with other measures. The authors evaluated 46 children aged from 5 to 7 years old. Twenty-two children had a diagnosis of PD and 24 had normal speech development. Auditory processing was assessed with diotic tests (localization of sound, verbal sequence memory, and non-verbal sequence memory), monotic (ear-specific) tests using the Pediatric Speech Intelligibility (PSI) Test, the Speech-in-Noise Test, and Dichotic Listening Tests with the Staggered Spondaic Word (SSW) test. The dichotic digit test performances in children with PD were highly compromised. Children with phonological disorders encountered difficulties in auditory analysis and synthesis, auditory segregation, temporal ordering, and memory. In general, these children were unable to separate the acoustic signal by using selective attention. These abilities are relevant to phonological acquisition and phoneme-grapheme associations; hence, children with PD may have difficulties in literacy acquisition. 

Murphy et al. [55] evaluated auditory and visual sustained attention in children with SSD. Fifty-five children participated in this study. Eighteen children were diagnosed with SSD, and thirty-seven were typically developing children. All children completed non-verbal auditory perceptual measures (Frequency Pattern Test and Gap in Noise Test), language tests (Picture Naming and Repetition of Words), a short-term memory test (the Digit Span Test), and a non-verbal IQ test (the RAVEN Test of Coloured Progressive Matrices). The outcome measures included the number of targets detected (HITS), the number of false alarms (FA), and the response times (RT). The children with SSD performed significantly poorer on both language tests, the short-term memory test, and both auditory perceptual tests. In general, the SSD group had poorer sustained attention in auditory-only stimuli as indicated by the proportion of HITS. Additionally, this group presented with more FA and longer reaction times than their normal-developing peers. The authors of this study discussed how nonverbal auditory perceptual impairments may contribute to auditory discrimination impairments among children with SSD. The findings related to increased false alarms may postulate that children with SSD take on anticipatory strategies that lead to stimuli leading to increased false alarms. Moreover, the decline in HIT performance suggests that increased tasks with auditory stimuli may lead to greater cognitive load and listening fatigue among children with SSD. The results of this study suggest that auditory processing abilities and phoneme discrimination have an effect on speech-sound assimilation and use.

### 3.4. Temporal Processing Deficits in Children with SSD

Jung and Lee [42] assessed temporal processing in 30 children (aged 8–11 years old) divided equally into three groups: (1) typically developing children, (2) children with SSD, and (3) children with SSD and cognitive difficulty (CD). The authors compared performance on auditory temporal resolution between the three groups based on the Gaps in Noise test (GIN). They found that the mean scores on the GIN test were significantly lower for children with SSD or CD compared to their age-matched typically developing peers. Furthermore, the IQ data obtained from the Weschler Intelligence Scale for Children III (WISC-III) explained a decent amount of variance in the GIN scores across children. The implications of this study reveal that children with deficient processing of rapid temporal cues in speech sound may experience deficits in phonological and phonemic information processing and intellectual functioning.

Jain et al. [33] included 32 children (aged 6–11 years) in their study, equally divided into two groups. The first group included typically developing children (TD), and the second group had children diagnosed with SSD. They investigated temporal resolution (TR), the time the auditory system takes to discriminate two acoustic stimuli, and temporal ordering (TO), the ability of the auditory system to process the sound in its order of occurrence. The Gap Detection Test (GDT) was used to assess TR and the Duration Pattern Test (DPT) was used to assess TO. Moreover, both groups of children were evaluated on phonological awareness tasks (blending, segmentation, syllable oddity, syllable deletion, phoneme deletion, and manipulation). As expected, children diagnosed with SSD performed significantly worse on phonological awareness tasks, The scores of phoneme oddity did not differ among the two groups. The authors found a significant difference in scores on the GDT and DPT between the groups. These results indicated that TD children had significantly higher scores for temporal processing than children with SSD. A negative correlation was obtained between GDT and the syllable deletion task and the phoneme deletion task, indicating that children with a lower GDT threshold performed better in syllable and phoneme deletion tasks. Furthermore, there was a significant positive correlation of the DPT with syllable oddity task and phoneme oddity task, indicating that children who had better scores in DPT performed better in syllable and phoneme oddity tasks. The findings agree with the literature, supporting a close relationship between temporal processing abilities and phonological awareness and, hence, reinforcing the importance of taking both processes into account when coming across children with SSD.

Vilela [56] conducted a pilot study to investigate the performance of the Pitch Pattern Sequence Test (PPS) and Duration Pattern Sequence Test (DPS) in children with phonological disorders following rehabilitative training focused on temporal processing. Fifteen children between ages 7 and 10 years old were divided into three groups: (1) children with SSD without APD without follow-up intervention (control group), (2) children with SSD and APD following formal auditory training, and (3) children with SSD and APD following informal auditory training. Group differences were not found in mean scores in the PPS and DPS task between the children receiving formal training and informal training; however, the performance significantly improved compared to children in the control group who did not receive any intervention. While there were no significant differences in test scores following different forms of intervention, this study suggests that children with SSD and APD can benefit from aural rehabilitation to improve their temporal processing abilities. 

### 3.5. Factors Affecting SSD and APD Severity

Barrozo et al. [46] tested twenty-one children diagnosed with SSD, eleven of whom were also diagnosed with APD. Children were aged between 7.0 and 9.11 years. The authors investigated the relationship between phonological and metalinguistic processing in children with SSD concerning APD. Phonological awareness was evaluated with particular emphasis on liquid simplification (LS), cluster reduction (CR), final consonant deletion (FCD), stop devoicing (SD), and fricative devoicing (FD). The evaluation was assessed using the Percentage of Consonants Correct (PCC), the Process Density Index (PDI), and alliteration of the Phonological Sensitivity Test (PST). Children with SSD and APD had lower speech-sound perception and production scores than those with SSD alone. The percentage of poor results was more significant in this group in all PST tasks. Furthermore, children with SSD and APD showed a higher occurrence of the phonological process of CR and more incredible difficulty in rhyme and alliteration tests. Cluster reduction was most frequently observed amongst all children, regardless of disorder; however, the occurrence was significantly worse amongst children with APD and SSD, suggesting that APD can hinder the phonological organization of complex structures. It is important to note that, in this study, SSD severity was higher in subjects with SSD and APD. The authors concluded that, amongst all the indices measured to characterize SSD severity, PDI was the best tool to differentiate children with SSD and APD from those with SSD alone. A PDI cutoff value of ≥0.54 was determined to sufficiently differentiate children with SSD and APD from those with SSD alone in their pool of subjects. Pereira and colleagues [37] reported additional implications from this study in their systematic review, including the following discussion points: (1) effects on temporal processing deficits in children with phonological disorders and (2) potential gender effects based on male prevalence in the study group.

Vilela et al. [47] compared the phonological, audiological, and central auditory processing abilities of two groups of children ages 7–10 years old: (1) children with SSD and no APD (*n* = 13) and (2) children with SSD and APD (*n* = 14). Percentage of Consonants Correct-Revised (PCC-R) scores were derived from phonological tests on picture-naming and imitation-of-word tasks, in addition to auditory processing skills focusing on auditory closure, binaural integration, and temporal ordering. The results of this study indicated a significant difference in the distribution of PCC-R values for both phonological tasks according to some impaired auditory skills. Furthermore, performance on binaural integration tasks was most strongly associated with SSD severity, suggesting temporal lobe deficiencies in children with SSD. It was also noted that performance on the phonological processing tasks was significantly lower amongst children who had SSD and APD, indicating that greater SSD severity is associated with higher chances of having APD. Vilela et al. [47] concluded a cutoff value based on the PCC-R to determine the likelihood of a child having SSD and APD. These findings suggest that below the two cutoff values (<83.4% and <84.5% for picture-naming and imitation-of-words tasks, respectively), the child may have an APD associated with the SSD. 

**Table 3:** Data from the language assessment.

Study	Country	StD	PT and G	M (SD)	PCC-RI AvsB	PCC-RN AvsB	PDI	PA	RAN	MEM	RL	EL	WARP
Sharma et al. [36]	Australia	OΒ	Total *n*: 68 A: no APD: 8 B: APD and LI: 39 C: APD and Li and RD: 32	7–12 9.7 ± 1.4 9.9 ± 1.7 9.7 ± 1.7	NR	NR	NR	NR	NR	NR	85.9 ± 19.3 81.4 ± 13.9 80.2 ± 13.2	84.3 ± 17 80.3 ± 12.4 80.5 ± 13	64 ± 54.3 77.3 ± 45.2 70.8 ± 41.4
Allen and Allan, [45]	Brazil	OΒ	Total *n*: 63 A: noAPD: 40 B: APD: 23	7–17	NR	NR	NR	*p* = 0.001	*p* = 0.012	*p* < 0.0001	*p* = 0.596	*p* = 0.762	*p* = 0.001
Barrozo et al. [46]	Brazil	OΒ	Total *n*: 21 A: CAPD: 11 B: no CAPD: 10	7.0–9.11 8.5 7.10	*p* = 0.031	*p* = 0.014	*p* = 0.007	NR	NR	NR	NR	NR	NR
Vilela et al. [47]	Brazil	OΒ	Total *n*: 27 A: SSDs NO CAPD: 13 B: SSDs AND CAPD: 14	7–10.11 8.8 ± 1.0 8.1 ± 0.7	*p* = 0.009	*p* = 0.012	NR	NR	NR	NR	NR	NR	NR
Lam et al. [43]	Australia	OΒ	Total *n*: 16 A: APD AND SSD: 8 B: APD no SSD: 8	7.5–10.2 9.08 ± 1.2 8.86 ± 0.79	NR	NR	NR	*p* = 0.012	*p* = 0.104	*p* = 0.175	NR	NR	*p* = 0.013

NR means no report and OB observation.

**Table 4:** Data from the behavioral assessment.

Study	Country	StD	PT and G	M (SD)	CHAPS	NVIQ (TONI-3 and RAVEN)	VOCAM	SENT. COMP
Sharma et al. [36]	Australia	OΒ	Total *n*: 68 A: no APD: 8 B: APD and LI: 39 C: APD and LI and RD: 32	7–12 9.7 ± 1.4 9.9 ± 1.7 9.7 ± 1.7	NR	112 ± 17.3 97.7 ± 13.1 97.6 ± 13.7	NR	NR
Allen and Allan, [45]	Brazil	OΒ	Total *n*: 63 A: no APD: 40 B: APD: 23	7–17	NR	*p* = 0.466	*p* = 0.02	*p* < 0.001
Barrozo et al. [46]	Brazil	OΒ	Total *n*: 21 A: CAPD: 11 B: no CAPD: 10	7.0–9.11 8.5 7.10	NR	NR	NR	NR
Vilela et al. [47]	Brazil	OΒ	Total *n*: 27 A: SSDs NO CAPD: 13 B: SSDs AND CAPD: 14	7–10.11 8.8 ± 1.0 8.1 ± 0.7	NR	NR	NR	NR
Lam et al. [43]	Australia	OΒ	Total *n*: 16 A: APD AND SSD: 8 B: APD no SSD: 8	7.5–10.2 9.08 ± 1.2 8.86 ± 0.79	*p* = 0.079	*p* = 0.254	*p* = 0.414	*p* = 0.36

NR means no report and OB observation.

**Table 5:** Data from the auditory processing assessment.

Study	Country	StD	PT and G	M (SD)	FI	DD	PPS	DPS	PST-A	PST-A vs. PST-V	ATTN	RT	APD and Memory	APD and Attention	APD and LR	APD and LE	GIN
Sharma et al. [36]	Australia	OΒ	Total *n*: 68 A: no APD:8 B: APD and LI: 39 C: APD and LI and RD: 32	7–12 9.7 ± 1.4 9.9 ± 1.7 9.7 ± 1.7	NR	NR	NR	NR	NR	NR	NR	NR	*p* = 0.465	*p* = 0.202	*p* = 0.002	*p* = 0.194	NR
Allen and Allan, [45]	Brazil	OΒ	Total *n*: 63 A: noAPD: 40 B: APD: 23	7–17	NR	*p* = *0.952*	RE: 95 ± 5.7 67.8 ± 22.3 LE 93.5 ± 6.6 67.4 ± 23.1	NR	NR	NR	NR	NR	*p* = 0.002	*p* < 0.001	NR	NR	NR
Barrozo et al. [46]	Brazil	OΒ	Total *n*: 21 A: CAPD: 11 B: no CAPD: 10	7.0–9.11 8.5 7.10	NR	NR	NR	NR	A: *p* = 0.772 Β: *p* = 0.504	*p* = 0.095	NR	NR	NR	NR	NR	NR	NR
Vilela et al. [47]	Brazil	OΒ	Total *n*: 27 A: NO CAPD: 13 B: CAPD: 14	7–10.11 8.8 ± 1.0 8.1 ± 0.7	A = 4 B = 13	A = 0 B = 13	A = 0 B = 9	A = 1 B = 8	NR	NR	NR	NR	NR	NR	NR	NR	NR
Lam et al. [43]	Australia	OΒ	Total *n*: 16 A: APD AND SSD: 8 B: APD no SSD:8	7.5–10.2 9.08 ± 1.2 8.86 ± 0.79	NR	NR	NR	NR	NR	NR	*p* = 0.104	*p* = 0.249	NR	NR	NR	NR	NR
Jung and Lee, [42]	South Korea	OB	Total *n*: 30 A:TD: 10 B: SSD: 10 C:CD: 10	8–11	NR	NR	NR	NR	NR	NR	NR	NR	NR	NR	NR	NR	MPCS 64% ± 3.1 54.2% ± 3.3 48.9% ± 4.6
Assis et al. [41]	Brazil	OB	Total *n*: 46 A: TD: 23 B: SSD: 23	4–8	NR	NR	NR	NR	NR	NR	NR	*p* = 0.04	NR	NR	NR	NR	MPCS 64% ± 3.1 54.2% ± 3.3 48.9% ± 4.6

NR means no report and OB observation.

## 4. Discussion

This systematic review investigated auditory perception and processing abilities, language skills, metalinguistic skills, and cognitive performance of children with SSD and APD and probed the possible comorbidities of APD and SSD. In summary, seven were comprehensively reviewed for this systematic review. The qualifying articles contained data on participants under the age of 18 years old and focused on children with suspected or clinical APD and children diagnosed with SSD. SSD can be clinically diagnosed as early as three years old while APD assessments can be reliably conducted at seven years of age and older. Hence, there is a significant interval of time that must pass for a child to be considered for an APD evaluation following SSD diagnosis, which might have contributed to the paucity of qualifying studies. Among children with SSD, there are individuals who exhibit phonological deficits deriving from auditory processing difficulties (e.g., auditory discrimination, auditory memory, etc.). SSD intervention at an early age may remediate clinical signs of phonological disorders. However, the auditory processing deficits contributing to altered perceptual phonological representation may persist, leading to lengthy, and less effective intervention outcomes [27,57]. To clarify whether maturation and experience help improve the clinical characteristics of APD and SSD, we would need longitudinal studies to report outcomes of SSD and APD post-puberty and into adulthood. 

To identify the likelihood of a child having APD and SSD, as well as to quantify the severity of SSD with the presence of APD, researchers have proposed several metrics [46,47]. These indices may provide insight to the clinician about disorder progression and expected developmental outcomes. There is an extensive literature indicating that auditory, language, and reading dysfunction are difficult to parse out due to overlapping disorder phenotypes and the lack of adequate diagnostic tools [36,58,59]. A recent review [60] has shown that, although questionnaires can separate children with APD from controls quite effectively, the power effect was smaller when trying to investigate APD in children with other language difficulties [60]. Hence, identifying appropriate assessment material to effectively determine children with APD and SSD was the second aim of this review. Tests used for the evaluation and differential diagnosis of the two disorders have been acquired from several studies reviewed in this paper [33,42,43,45,58,61,62]. This discussion proposes the implementation of test measures assessing three areas of auditory processing: (1) frequency discrimination, (2) binaural integration, and (3) temporal processing. Assessing these processes may benefit clinicians in specifying the diagnostic assessment of SSD and streamlining appropriate intervention when auditory processing deficits are present.

Phonological awareness is critical to literacy development [63,64,65]. Researchers found that the auditory perception, language, and literacy abilities of children with APD are deficient [32,42,60]. Through our reviewed studies, children with SSD and APD have the compromised skills necessary for the use of language to understand and monitor oral and written communication [43,44,56]. Frequency discrimination (FD), the ability to detect frequency differences between tones or sounds, is one of the various AP deficits linked to poor reading. Overall, the literature has documented an association between FD performance and phonological awareness and non-lexical reading [66,67,68,69,70,71,72]. Given that non-lexical reading and phonological awareness are critical to learning letter–sound correspondences, it is evident that FD, as a component of auditory processing, is an essential area to formally evaluate in children with SSD. Parallel to this matter, children with similar profiles of APD may or may not have language impairments (LI) as indicated by Sharma et al. [44]. Therefore, it is impossible to predict the presence of LI in children with APD based on the APD test battery alone. 

Several studies have shown that children with SSD exhibit difficulty in discriminating, sequencing, or remembering brief stimuli in rapid succession [65,66]. Across the literature, auditory temporal processing has been found to be a significant component contributing to these difficulties. Temporal processing plays a crucial role in sound perception and phonological awareness owing to its role in perceiving rapid acoustical changes in speech stimuli [24]. The Gap Detection Test (GDT) is used in auditory processing assessment to evaluate temporal processing abilities. APD due to temporal processing deficits may be defined by poor decoding ability (e.g., impaired writing, phoneme analysis and synthesis difficulties, and difficulty integrating acoustic aspects of speech) and/or organization of auditory information (e.g., difficulty organizing acoustic events in time and ordering speech sounds). Hence, the GDT performance can be clinically used to further evaluate the phoneme-specific speech perception of children with SSD. It may also be considered in planning a non-linguistic auditory intervention. 

Binaural integration is an auditory processing skill that allows an individual to fuse different auditory inputs from both ears into a meaningful stream of input. This process is often assessed through the Dichotic Digits Test (DDT). The DDT has been used in diagnostic test batteries to identify lesions of the left temporal bone. This area of the brain plays a vital role during the various stages of developing speech perception and production. The association between SSD severity and poor linguistic sound-processing performance in dichotic listening tasks such as DDT strongly indicates temporal lobe deficiencies in children with SSD [47]. This finding may provide great value for clinicians to utilize a dichotic listening task when assessing phonological performance to identify SSD disorder severity and strengthen auditory skills during the therapeutic intervention, based on assessment performance. This review has shown that APD assessment should be considered in children with SSD to determine which auditory skills are most affected. 

### Limitations of the Current Review 

Several limitations in this study must be considered. First, the included articles were published in the English language. Therefore, studies that reported in other languages were not included, which could be a disadvantage for this work, as it narrows down the findings of the available literature globally. The inclusion of articles reporting assessment with behavioral measures in this review may also be a limiting factor in the overall discussion of APD/SSD pathophysiology assessment. Electrophysiological measures are an integral part of a complete AP assessment protocol. Our recommendation to assess specific AP characteristics in children with SSD supports the use of electrophysiological tests. In addition to the chronological gap between SSD and APD diagnosis, the low number of articles reviewed in this systematic review is also due to the limited coherence between auditory and phonological awareness disorders in the literature. Most of the work on phonological awareness focuses on early diagnosis for preschool children and early intervention programs, whereas auditory processing assessment occurs at school age. It is important to consider the age of neural maturation in evaluating performance on AP tests as the age of testing has been shown to positively correlate with performance outcomes, as discussed by the Pereira et al. [38] group. The low coherence in the results of qualifying studies would necessitate an analysis of moderating factors, such as age, gender, comorbidity, cognitive abilities, executive functioning, and task type. The low number of qualifying articles precluded this investigation. An analysis of moderating factors would be a necessary step in future studies when the research in this area provides sufficient studies [73]. 

## 5. Conclusions

The difficulties of children with APD are multimodal. The effectiveness of treatment is directly related to the specificity of the diagnosis. Multidisciplinary assessments that include language, literacy, cognition, attention, memory, and auditory processing measures (consisting of both non-speech and speech tests) are needed to specify how auditory processing disorder affects language development. Adapting quantitative indices from testing material, as suggested by several authors in this review, may be helpful in this population to predict language outcomes and disorder severity. Due to the complex nature of APD, a multidisciplinary approach to the screening process is essential when evaluating children with SSD. Each interdisciplinary team member should be responsible for gathering information in their area of expertise. A thorough evaluation with cognitive and psychoeducational instruments is necessary to delineate cognitive and academic strengths and weaknesses and to detect confounding disorders. Detecting comorbidity and describing appropriate assessment tests for both APD and SSD is an integrative process for audiologists, speech pathologists, educational specialists, and supporting caregivers. It is essential to note that all studies recommend evaluating acoustic processing in children seven years of age and older, as more precise and consistent results are recorded, while speech-sound disorders are recorded at a much younger age. Future research should develop evaluation procedures that can cope with the difficulty of evaluating age-dependent normative data with reliability, efficiency, and validity.
